# Responsive and intelligent service recommendation method based on deep learning in cloud service

**DOI:** 10.3389/fgene.2022.966483

**Published:** 2022-11-22

**Authors:** Lei Yu, Yucong Duan

**Affiliations:** ^1^ Department of Computer Science, Inner Mongolia University, Hohhot, China; ^2^ Department of Data Science and Big Data Technology, Hainan University, Haikou, China

**Keywords:** deep learning, QoS prediction, service recommendation, services, intelligent

## Abstract

The rapid expansion of the cloud service market is inseparable from its widely acclaimed service model. The rapid increase in the number of cloud services has resulted in the phenomenon of service overload. Service recommendations based on services’ function attributes are important because they can help users filter services with specific functions, such as the function of guessing hobbies on shopping websites and daily recommendation functions in the listening app. Nowadays, cloud service market has a large number of services, which have similar functions, but the quality of service (QoS) is very different. Although the recommendation based on services’ function attributes satisfies users’ basic demands, it ignores the impact of the QoS on the user experience. To further improve users’ satisfaction with service recommendations, researchers try to recommend services based on services’ non-functional attributes. There is sparsity of the QoS matrix in the real world, which brings obstacles to service recommendation; hence, the prediction of the QoS becomes a solution to overcome this obstacle. Scholars have tried to use collaborative filtering (CF) methods and matrix factorization (MF) methods to predict the QoS, but these methods face two challenges. The first challenge is the sparsity of data; the sparsity makes it difficult for CF to accurately determine whether users are similar, and the gap between the hidden matrices obtained by MF decomposition is large; the second challenge is the cold start of recommendation when new users (or services) participate in the recommendation; its historical record is vacant, making accurately predicting the QoS value be more difficult. To solve the aforementioned problems, this study mainly does the following work: 1) we organized the QoS matrix into a service call record, which contains user characteristic information and current QoS. 2) We proposed a QoS prediction method based on GRU–GAN. 3) We used the time series data for quality predictions and compared some QoS prediction methods, such as CF and MF. The results showed that the prediction results based on GRU–GAN are far superior to other prediction methods under the same data density. We aim to help the engineering community promote their findings, shape the technological revolution, improve multidisciplinary collaborations, and collectively create a better future.

## 1 Introduction

**FIGURE 1:**
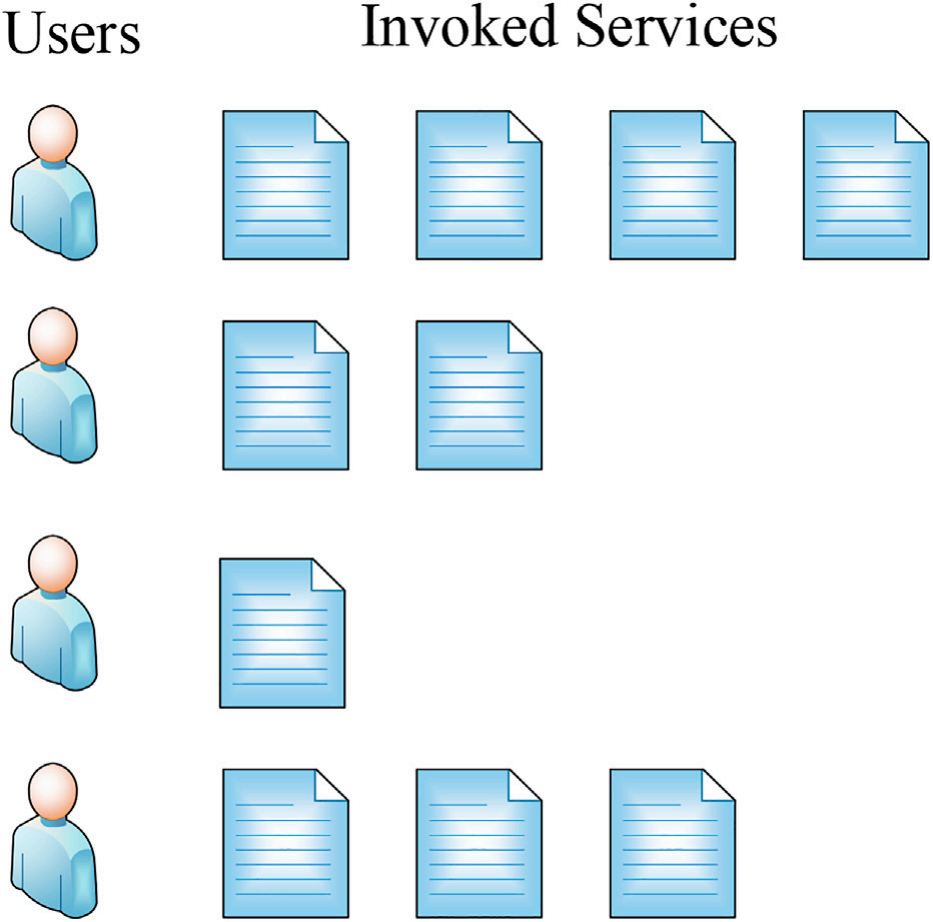
Recommendation scenario.

While massive web services bring convenience and innovation, the coverage of web service resources is getting wider and faster (Zhang Y. et al., 2020). The scale of the cloud service market in the future should not be underestimated. The explosive growth of the number of web services in the cloud service community has become a new challenge because it is difficult for users to select high-quality services from many services. In recent years, more and more people try to use deep learning (DL) models (Nath and Wu, 2020) to learn the functional properties of web services, such as service operations, inputs, outputs, and prerequisites, to realize user demand for functionality. Its advantage is that it can select services with specific functions in a targeted manner according to the specific functions of the services required. This technology is now relatively mature. However, existing research has found that when there are a large number of services with the same or similar functions, filtering only based on the functional attributes of the services is less effective. People began to try to introduce QoS to measure the non-functional performance of a service. The study found that although QoS-based service recommendations can recommend higher quality services for people, the sparseness of real-world data makes the recommendation results unreliable for users. Therefore, people try to use the quality prediction of web services (hereinafter referred to as quality prediction) to solve the problem caused by data sparse. Quality prediction is an essential link in service recommendation; however, most quality predictions now use collaborative filtering (CF) and matrix factorization (MF) methods. However, these two types of methods only use the interaction information between the user and the service as the main basis, ignoring the user’s personalized feature information (Hamilton, 2022). Therefore, when faced with the cold start problem, the reliability of the predicted results is low due to the lack of the aforementioned interactive information. Figure 1 shows a service recommendation scenario where service users used a list of services and intend to use more suitable services by automatic recommendations.

Based on the aforementioned problems, this study rearranged each call record to include the feature information of the user under the service and uses a deep neural network to analyze the nonlinear relationship between the feature information and response time in each record so that the QoS prediction value is reliable. The scale of the cloud service market is growing rapidly (Sandhu, 2021). Although it brings more choices to users, the dazzling array of services also makes users dazzled. Cloud service recommendation can effectively solve this problem, so the main work of this study is to use deep learning to research cloud service recommendations and explore more accurate cloud service recommendation methods to recommend services with higher QoS values to users. The purpose of this subject is to make its contribution in the field of service recommendation, which has certain academic value and research significance. With the promotion of personalized service concepts and the wide application of service-oriented computing (SOC), more and more enterprises provide users with personalized products. Service-oriented architecture (SOA) is the most recognized implementation, which makes service invocation more convenient. Therefore, there is explosive growth in the number of web services (WSs), and it is difficult for conventional service recommendation methods to efficiently process and utilize services (Zhang W. et al., 2020). This phenomenon is also called service overload. People try to use the service recommendation method to solve the problem of service overload. Service recommendation is mainly divided into two recommendation methods based on function and non-function. Among them, the technology of mining users’ functional requirements is relatively mature. How to filter service recommendation methods with higher service quality became a hotspot in the field of service recommendation. To solve our problems, this study mainly did the following work:

1) We organized the QoS matrix into a service call record, which contains user characteristic information and current QoS. This study used the public dataset WS-Dream as experimental data and found that there are a large number of irrelevant and redundant information in the dataset, so we used a variety of tools to extract feature information, specifically including filtering fields, filling missing values, morphological restoration, and converting lowercase, and then performing feature extraction to generate time series data.
2) We proposed a QoS prediction method based on GRU–GAN. Compared with other service recommendation methods, this method can overcome the drawbacks of linear operations brought by collaborative filtering-based service recommendation methods and matrix factorization-based service recommendation methods by learning the nonlinear relationship between eigenvalues in time series data. Through the generative adversarial network, the real-time series data and the predicted time series data are used for adversarial training to improve the prediction performance of the model.
3) We used the time series data for quality prediction and compared some QoS prediction methods, such as CF and MF. The results show that the prediction results based on GRU–GAN are far superior to other prediction methods under the same data density.

At present, there are roughly three solutions for QoS-based service recommendation: CF-based service recommendation (Lo et al., 2012a), MF-based service recommendation (Lo et al., 2012b), and DL-based service recommendation (Hudson et al., 2021). The CF-based method is an ancient algorithm in the field of service recommendation. The main idea of the algorithm is to use interaction records to find similar users (or services) and to recommend services for users based on the view that similar users have similar evaluations of services. CF-based methods are further subdivided into two service recommendation methods: memory-based and model-based methods. Among them, memory-based methods can be subdivided into three types, which are user-based (Shao et al., 2007), item-based (Deshpande and Karypis, 2004), and a combination of the two (Zheng et al., 2009). The memory-based CF method calculates the similarity between users (or services) according to the matrix of users calling services, then predicts the QoS of similar users (or services) based on the similarity, and then sorts the QoS in a certain order according to the prediction results, and finally selects the top K services with the best service quality and recommend them to users. Wu et al. (2012) proposed a neighborhood-based CF method, which eliminated different levels of the QoS by adjusting the similarity calculation method, and then used a similarity fusion method to reduce the impact of data sparsity. To improve prediction accuracy, scholars have begun to pay attention to the impact of contextual information such as time and space on the QoS. For example, Yu and Huang (2014) proposed a time-aware CF algorithm to predict the missing QoS; Liu et al. (2015) used a spatially aware CF algorithm to improve the performance of service recommendations. However, when the aforementioned methods were not able to provide real-time recommendations when faced with a large amount of data, Zhang et al. (2011) proposed the WSPred model to improve prediction accuracy by embedding temporal information. Although CF has achieved more intentional results in the field of service recommendation in the early stage (Wu et al., 2012; Liu et al., 2015), there are still the following drawbacks: 1) data sparsity: CF methods mainly rely on the call records between users and services to calculate similarity; these call records usually only provide some low-dimensional and linear features, so when the data density is small, insufficient learning of features limits the improvement of prediction performance. 2) Cold start: when a new user (or service) is the target user, the reliability of the similarity calculation result is low.

To solve the interference caused by the aforementioned problems, the MF method has been applied to service recommendations by many scholars. For example, there is a QoS matrix $R_{m*n}$ generated by a user calling service, and the “user implicit matrix $U_{m*k}$ ” and “service implicit matrix $S_{k*n}$ ” are obtained through MF. These two matrices are used to describe the characteristics of users and services, respectively. By optimizing the objective function to make the product of the “hidden matrix” closer to R, the missing data in R are also filled.

Tang N. et al. (2016) proposed a QoS prediction algorithm ClustTD based on location clustering and tensor decomposition. This method uses location information to cluster users and services, and then performs tensor decomposition on the user and service vectors. The results are weighted and combined to finally obtain the predicted value of the QoS; Xu et al. (2013) used the upper and lower information of the service and user location to perform matrix decomposition, and proposed LE-MF for the prediction of missing values, and user clustering and service clustering, reduce the volume of the QoS matrix, and finally complete the prediction task of vacancy values through matrix decomposition; Yin et al. (2016) considered the impact of the network environment on the QoS, and combined the autonomous system into the judging network location neighbor index. A QoS prediction method based on network location-aware neighbor selection web service recommendation was proposed, which improved the prediction performance by reducing the solution space; Qi et al. (2020) considered from the perspective of service security and concluded that although the spatiotemporal information of users and services improved, it improves the reliability of the recommendation but also reduces the security, so they add the location-sensitive hashing technology to the space-time information to enhance the privacy protection of users and services. Matrix factorization improves the reliability of quality prediction results by alleviating the problem of data sparsity, but the number of features involved in the calculation is limited, which makes it difficult to overcome the challenges brought by a cold start.

## 2 Related work

In SOA recommended systems (Nitu et al., 2021), the user’s personalized needs are presented in the form of QoS (Tran and Tsuji, 2009). QoS is an important indicator for evaluating service performance, so it can be used as the most important factor to distinguish the quality of web services. QoS includes performance, reliability, security, and some other metrics. For users, the level of the QoS value determines the QoS experience; for services, the level of the QoS value indicates the quality of service performance and also affects the popularity of the service.

Deep learning (DL) is an important subcategory of machine learning, which trains and captures data features through neural networks (Li et al., 2020). DL learns the abstract expression of the data and the inherent laws between the data in the massive data, and extracts the feature representation of the complex level from it. Through the aforementioned process, the computer has the ability to analyze and learn like a human. The early neural network is similar to the combination of simple neural units to form an artificial neural network. Since the DL model is widely recognized, various sub-models are also derived, such as the convolutional neural network (CNN) model, deep belief network (DBN) model, belief network(BN) model, and stacked autoencoder (SAE) model (Zhang et al., 2021).

The core of the RNN analysis problem is to find the invisible connection between the input time series data. The RNN is used to process time series data, and the effective information contained in time series data at different times is different, so the RNN can be regarded as a kind of a neural network with short-term memory ability.

In the RNN, the current neuron can accept the information of not only its previous and backward neurons but also its own information, and finally form a network structure with loops. Because of the characteristics of receiving neurons, the RNN has a stronger memory ability. At present, RNNs have been widely used in tasks such as computer vision, meteorology, and text sentiment classification. However, due to the long training time of the original RNN, the training will cause the gradient to be in two extreme states, that is, explosion and disappearance.

Gated recurrent units (GRUs) are a variant of LSTM. The structure of LSTM is relatively complex, and the number of parameters it contains far exceeds that of GRU, which makes the training difficulty of parameters sharply increased. In response to the aforementioned situation, the GRU was proposed to reduce the number of parameters in LSTM and ensure the effect of training. The specific method is that the GRU combines the forgetting gate and the input gate to reduce the complexity of the neural network, which not only ensures the memory ability of the RNN but also reduces the complexity of the neural network. It improves the training efficiency of the network. The GRU contains two gates. The update gate determines the degree to which the information in the previous time series data is brought into the future, and the larger the value, the greater the degree of introduction; the reset gate determines the importance of the information in the current time series, and the larger the value is, the current time series data are less important (Qi et al., 2020a).

The GAN is a neural network that uses game thinking (Creswell et al., 2018). The GAN is mainly composed of generator G (Generator) and discriminator D (Discriminator). Generator G learns the distribution of the given data, and when the noise is input to the generator, it will generate “fake data” similar to the real sample; discriminator D mainly identifies “fake data” from a sample set that is a mixture of real and fake. G and D continuously update the loss function through adversarial training to achieve the overall optimization goal. Through multiple game processes, the generator can achieve the goal of “mixing the fake with the real.” The optimal state is achieved when discriminator D cannot distinguish the authenticity of the data, that is, when the output probability of discriminator D is 1/2.

## 3 Model design

**FIGURE 2:**
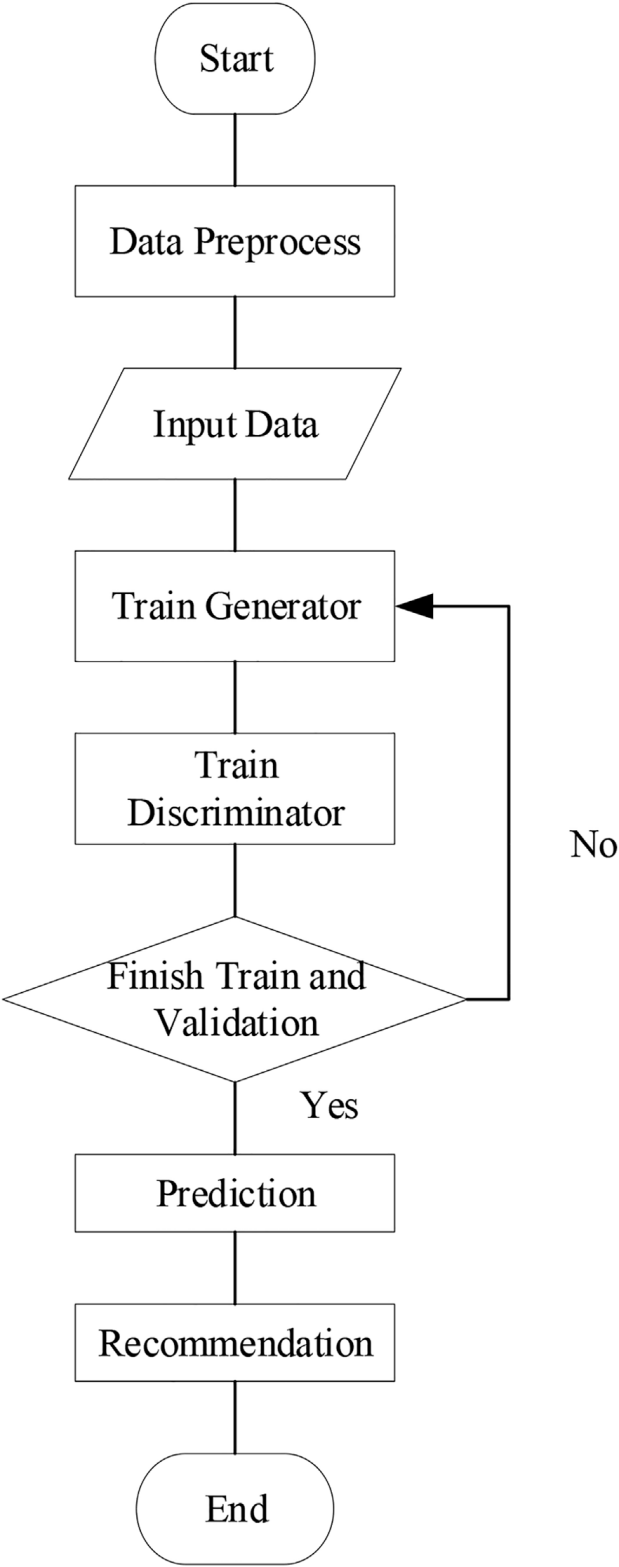
Methodological framework.

The user-service call records are generated through the real data set WS-Dream, and then a combination of the gated recurrent neural network and the adversarial neural network-based adversarial gated recurrent neural network (GRU–GAN) is proposed in this study to predict the motivation and value of the QoS-specific implementation process. This method can effectively predict the QoS value when the data sparsity is low and can also alleviate the impact of user cold start to a certain extent. Figure 2 shows our methodological framework.

### 3.1 Generator model

**FIGURE 3:**
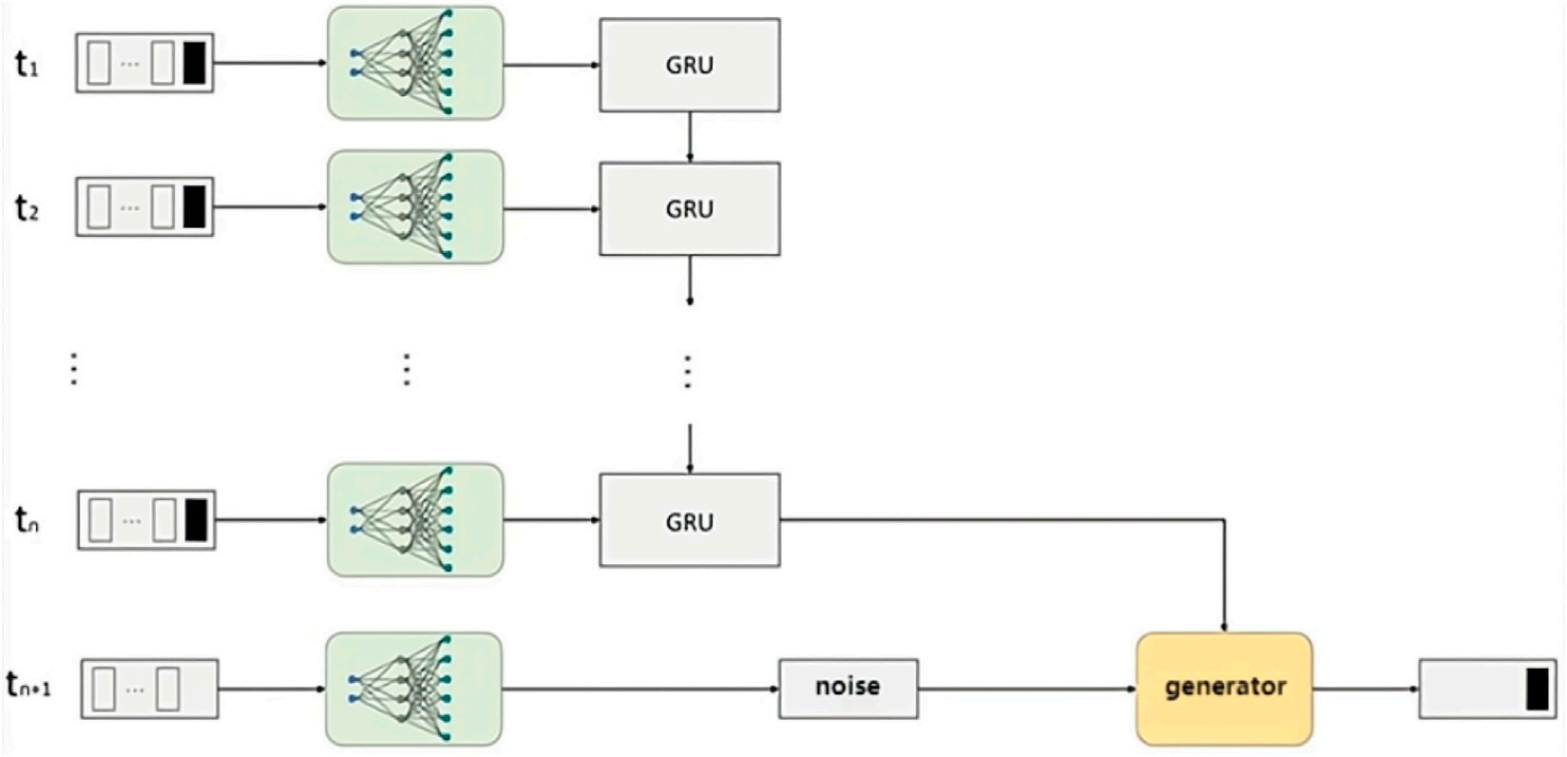
Generator model.

In the generative adversarial network, the design of the generator needs to be specifically designed according to specific experiments. In the training phase of the GRU–GAN model, the main purpose of G is to “cheat” D with the predicted QoS. The generator uses the time series within the 0-t time series to train the weight of each hidden neuron, and then whenever the noise data converted from the user feature information at the next moment are entered, it will predict the next moment when the user invokes the service. This section will introduce the specific design scheme and training process of the generator in the quality prediction model of this study. Figure 3 shows the generator model in the QoS prediction model proposed in this study.

The input of the generator is a time series and the output is the QoS prediction value at the future time. The QoS of the generator from input noise to output can be summarized as Eq. 1.

$$\hat{y_{u,t+1}}=G\left(t_{u,t},x_{u,t+1}\right),$$ (1)

where $\hat{y_{u,t+1}}$ represents the QoS of user u at time t+1, $t_{u,t}$ represents t real-time series generated by u users calling s services, and $x_{u,t+1}$ represents the feature information of user u in the t+1st time series. G () is to train the generator function using the time series at time 0-t, which contains the weights for each hidden neuron.

Since the data type used in this study are time series data, RNN itself is a kind of neural network suitable for studying time series data. In related research, it was found that GRU can better deal with the gradient decay problem of RNN and can better capture the relationship between time series. Considering the applicability of GRU in this study, this article puts GRU into the generator model. At the same time, to ensure the consistency of the data dimensions of each connected part, the fully connected neural network is also put into the generator model, and the Leaky ReLU activation function is used in the fully connected layer, where a = 0.02. In GRU–GAN, the loss function of the generator is defined as the error between the predicted value of the QoS and the real value of the QoS. This study uses the L1 loss function to measure the error. The loss function calculation formula of generator G is as shown in Eq. 2.

$${Loss}_{G}=\overset{n}{\underset{t=1}{\sum }}\left|y_{t}-\hat{y_{t}}\right|.$$ (2)

Among them, $y_{t}$ represents the real QoS value at time t and $\hat{y_{t}}$ represents the predicted QoS value at time t. By minimizing the loss function of G, the error between the real data and the predicted data can be reduced, thereby improving the prediction performance of the generator.

In the generator network, the real data set is first input in chronological order; then the fully connected layer is used to map the dimension of the real data to the same dimension as the input layer of the GRU network, and the distribution characteristics of each feature in the real data and the QoS characteristics are learned. The fitting process, which can be expressed as a regression equation, constructs a function from a historical variable to the current value of a variable in a certain dimension. This fitting process can be expressed as Eq. 3.

$$\hat{y_{t}}=\theta_{n}x_{n}^{t}+e_{t}.$$ (3)

Among them, $\hat{y_{t}}$ represents the predicted value of the QoS at time t, $\theta_{n}$ is the 1*n-dimensional weight vector at time t, $x_{n}^{t}$ is the feature vector of the n*1-dimensional user-service call record at the current time, and $e_{t}$ is the current time error.

Therefore, whenever the basic information of the current state of the user is input, Eq. 3 will calculate the QoS value of the service invoked by the user at the current moment. The more the training samples are input, the better the fitting effect of the function will be. The QoS will get closer and closer to the real QoS as the number of iterations increases. The forward training process and backpropagation process of GRU in this study will be introduced separately in the following section.

The reset gate determines how much of the previously input information is written on the candidate set. First, the product of the weight matrix $A_{r}$ and $h_{t-1}$ and $x_{t}$ spliced into a matrix is calculated, and then the gate to convert the calculation result of $A_{r}∙\left[h_{t-1},x_{t}\right]$ is reset between 0 and1 through the activation function. The larger the value of $r_{t}$ , the more information is written in the previous state. The calculation method of the reset gate is shown in Eq. 4.

$$r_{t}=\sigma \left(A_{r}∙\left[h_{t-1},x_{t}\right]+e_{r}\right),$$ (4)

where $A_{r}$ represents the weight matrix of the reset gate, $h_{t-1}$ represents the hidden state at time t-1, $x_{t}$ represents the sequence input at time t (through the fully connected layer), $e_{r}$ represents the bias of the reset gate, and σ is the sigmoid function.

The $r_{t}$ value calculated by Eq. 4 will be used for the calculation of the candidate hidden state $h_{t-1}$ ; tanh converts the calculation result of $W_{\tilde{h}}∙\left[r_{t}*h_{t-1},x_{t}\right]$ into a value between −1 and 1. From the calculation formula of $\tilde{h_{t}}$ Eq. 5, it can be seen that when $r_{t}$ is smaller, the smaller is $\tilde{h_{t}}$ , that is, more past information is needed.

$$\tilde{h_{t}}=\tanh\left(A_{\tilde{h}}∙\left[r_{t}*h_{t-1}{,x}_{t}\right]\right),$$ (5)

where $A_{\tilde{h}}$ represents the weight of the candidate hidden state and tanh represents the activation function.

The update gate $z_{t}$ determines the extent to which the state information at time t-1 is brought into the current state. The calculation of the update gate is similar to that of the reset gate, and its calculation method is shown in Eq. 6.

$$z_{t}=\sigma \left(A_{z}∙\left[h_{t-1},x_{t}\right]+c_{z}\right),$$ (6)

where $A_{z}$ represents the weight of the update gate and $c_{z}$ represents the bias of the update gate.

Based on the aforementioned calculation process, hidden state $h_{t}$ at the next moment can be obtained. In Eq. 7, it can be seen that when the value of $z_{t}$ is larger, memory data $z_{t}*\tilde{h_{t}}$ are more, and forgotten data $\left(1-z_{t}\right)*h_{t-1}$ are less.

$$h_{t}=\left(1-z_{t}\right)*h_{t-1}+z_{t}*\tilde{h_{t}}.$$ (7)

After completing the forward propagation process, we obtained relatively good neural network parameters. To optimize the parameters of the neural network, we need to optimize the weight parameters and bias parameters through backpropagation until the upper limit of the number of iterations is reached. At this time, ${Loss}_{G}$ is lower; that is, the performance of the generator network is better. The process of backpropagation will be described as follows.

$h_{t}$ obtained by Eq. 1 is $\hat{y_{t}}$ in Eq. 1, and minimizing the loss function of the entire QoS is the goal of the entire training period. The loss function definition at this time can be expressed as Eq. 8.

$$\left\{ \begin{array}{c} l\left(t\right)=\left|h_{t}-\hat{y_{t}}\right|\\ L=\overset{T}{\underset{1}{\sum }}\left(t\right) \end{array}\right.,$$ (8)

where l(t) represents the loss function value calculated at time t and L represents the cumulative loss of the entire time series during training.

### 3.2 Discriminator model

This section mainly introduces the discriminator model, including the main tasks, model structure, and processing process of the discriminator model. The main task of discriminator D is to distinguish true from false from the real data set and the predicted data set generated by the generator, and give a probability value between 0 and 1 to the records in input D. The loss function of D is shown in Eq. 9.

$${Loss}_{D}=\overset{n}{\underset{t=1}{\sum }}D\left(\hat{y_{t}}\right)-D\left(y_{t}\right).$$ (9)

**FIGURE 4:**
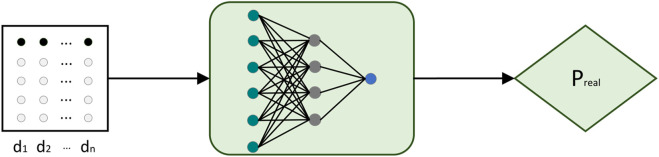
Discriminator model.

The discriminator mainly consists of three fully connected layers. The input time series is mapped to a probability value between 0 and 1 through a fully connected neural network. The larger the probability value, the more likely the current input time series is to be true. Otherwise, the input is considered to be more likely to be a predicted value. The structure of the discriminator network is shown in Figure 4.

### 3.3 GRU–GAN

In the first two sections, the generator model and the discriminator model are introduced, respectively. In this subsection, the whole of the proposed GRU–GAN-based QoS prediction model will be elaborated. In the initial GAN algorithm network, the generator will introduce random noise, and the random noise and the real data satisfy the same distribution and have similar probability densities. The discriminator integrates the input real samples and noise samples into a new sample collection and obtains the distribution probability between 0 and1 through the fully connected layer, that is, the probability that the sample includes the real data. The generator is responsible for feeding the data, and the discriminator is responsible for separating the data and using constant comparison to complete the balance to achieve learning. The input to the generator in a GAN is early data in the sequence, and the output is the predicted sequence data. Therefore, the input of the discriminator can be represented by two parts, which are the real-time series and the future time series obtained by the generator; the output of the discriminator is the probability distribution of these two kinds of data.

For the data set in this study, the data output by the generator need to meet the same distribution law as the real data, and the generated data are also the time series. The difference from the real data is the response time in the sequence.

In a general generative adversarial network, the generator converts a set of input noises into a fake sample set, and then through adversarial training, the predicted data generated by the generator have the same distribution as the real data. In this study, the output of the generator is the response time of a specific time series, so the output of the generator has a one-to-one correspondence with the input. Therefore, in the GRU–GAN model, the input random noise z is a specific set of time series data.

## 4 Experiment

### 4.1 Experiment data

This study conducts experiments on the WS-Dream dataset, which is widely used by academia to study QoS prediction problems. The dataset was originally collected by Zhang et al. (QoS values for 5,828 services from 339 distributed computers in PlanetLab’s 30 countries).

### 4.2 Evaluation criteria

To evaluate the performance of the QoS prediction model based on GRU–GAN, we choose the two most widely used metrics for continuous variables: mean absolute error (MAE) and root mean squared error (RMSE).

#### 4.2.1 MAE

MAE represents the mean of the absolute error between the predicted value and the observed value, and it represents the mean margin of error of the predicted value. It is also a commonly used regression loss function, and its calculation method is shown in Eq. 10.

$$MAE=\frac{\sum_{u,s}\left|r_{u,s}-\hat{r_{u,s}}\right|}{N}.$$ (10)

#### 4.2.2 RMSE

The formula for calculating the root mean square error is as follows:

$$RMSE=\sqrt{\frac{\sum_{u,s}{\left(r_{u,s}-\hat{r_{u,s}}\right)}^{2}}{N}}.$$ (11)

### 4.3 Contrast methods

For the prediction effect of this deep learning-based service recommendation model, this study selects some representative QoS prediction methods for comparison. These comparison methods are described in detail next.

1. UPCC (Shao et al., 2007): this method is a memory-based collaborative filtering algorithm that uses the Pearson coefficient to find similar users and uses the QoS values of similar users to predict the QoS value of the target user.
2. IPCC (Deshpande and Karypis, 2004): similar to method 1, this method looks for similar services and uses the QoS values of similar services to predict the QoS value of the target service.
3. UIPCC (Zheng et al., 2009): this method combines the advantages of UPCC and IPCC to predict QoS values, and add parameters to balance the roles of the two.
4. CMF (Koren, 2010): this method uses the classical matrix factorization method to build a global model for quality prediction.
5. NMF (Lee and Seung, 1999): although this method is also based on matrix decomposition to solve the QoS value, this method adds a non-negative factor to matrix decomposition to improve the reliability of matrix decomposition.
6. PMF (Mnih and Salakhutdinov, 2008): this method introduces a probability model for probability matrix decomposition and optimizes the original matrix decomposition model.
7. NIMF (Zheng et al., 2013): this method calculates N(u) through the Pearson coefficient and adds the user’s domain information to the matrix decomposition.
8. NAMF (Tang et al., 2016a): this method adds basic user information to matrix decomposition, filters domain users according to geographic location, and adds neighborhood information to matrix decomposition.
9. QoS prediction method based on GRU: this method is the reference experiment of this experiment. The difference between the two is that only the GRU network is included in method 9, while the method proposed in this study combines two neural networks: GRU and GAN; with the same point, because the input data of these two methods are the same, they can be used to predict the QoS value of the vacancy.

**FIGURE 5:**
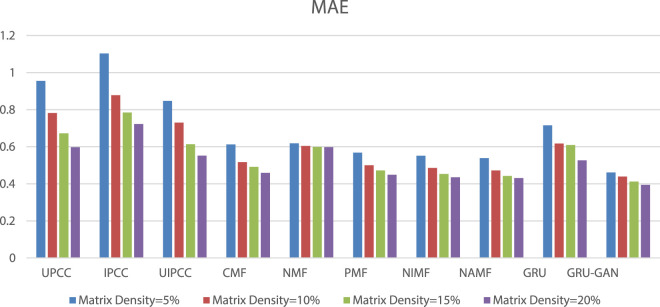
MAE comparison.

**FIGURE 6:**
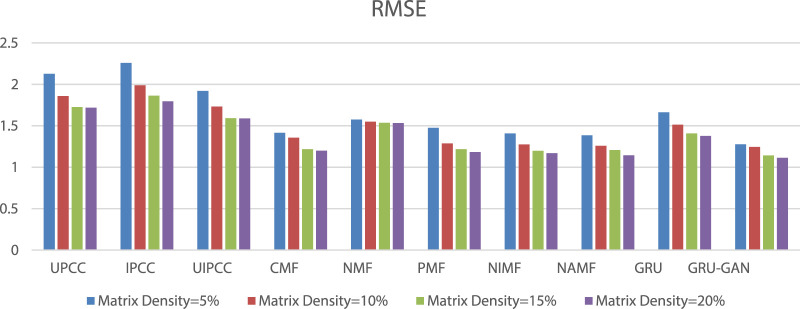
RMSE comparison.

Based on Figure 5 and Figure 6, we found that the prediction accuracy based on MF is better than that based on the CF method, and the GRU–GAN-based method proposed in this study has the prediction accuracy of MF. Comparing the mean values of MAE under the four densities, the values of models in this study are decreased with a range from 0.325 to 0.044 and lower than UPCC, IPCC, UIPCC, CMF, NMF, PMF, NIMF, NAMF, and GRU. The decrement of the average value of RMSE ranges from 0.78 to 0.3, and it is lower than the aforementioned 9 methods. Our method takes longer periods to achieve better prediction results because our model has more parameters to be trained to better fit the training data, which are the characteristics of GRU–GAN.

## 5 Conclusion

Although the recommendation based on the services’ function attributes satisfies users’ demands for service function, it ignores the impact of the QoS on the user experience. To further improve users’ satisfaction with service recommendations, people try to recommend services based on services’ non-functional attributes. There is sparsity of the QoS matrix in the real world, which brings obstacles to service recommendation; hence, the prediction of QoS becomes a solution to overcome this obstacle. Scholars have tried to use collaborative filtering (CF) methods and matrix factorization (MF) methods to predict the QoS, but these methods face two challenges. The first challenge is the sparsity of data; the sparsity makes it difficult for CF to accurately determine whether users are similar, and the gap between the hidden matrices obtained by MF decomposition is large; the second challenge is the cold start of recommendation when new users (or services) participate in the recommendation; its historical record is vacant, making accurately predicting the QoS value be more difficult. To solve the aforementioned problems, this study mainly did the following work: 1) we organized the QoS matrix into a service call record, which contains user characteristic information and current QoS. 2) We proposed a QoS prediction method based on GRU–GAN. 3) We used the time series data for quality prediction and compared some QoS prediction methods, such as CF and MF. The results showed that the prediction results based on GRU–GAN are far superior to other prediction methods under the same data density.

## Data Availability

The original contributions presented in the study are included in the article/Supplementary Materials; further inquiries can be directed to the corresponding authors.
